# Examining factors that influence medication adherence with children seen at outpatient department in Western China: a cross-sectional survey

**DOI:** 10.1038/s41598-023-43538-4

**Published:** 2023-10-05

**Authors:** Chunsong Yang, Yaya Yang, Lingli Zhang, Dan Li

**Affiliations:** 1 Department of Pharmacy, Evidence-Based Pharmacy Center, West China Second University Hospital, Sichuan University, No. 20,Third Section, Renmin Nan Lu, Chengdu, 610041 Sichuan China; 2 Key Laboratory of Birth Defects and Related Diseases of Women and Children (Sichuan University), Ministry of Education, Chengdu, China; 3 Department of Pediatric Clinic, West China Second University Hospital, Sichuan University, No. 20,Third Section, Renmin Nan Lu, Chengdu, 610041 Sichuan China

**Keywords:** Diseases, Health care, Medical research

## Abstract

We aimed to evaluate the prevalence of medication adherence, assess the association between guardians’ mental health and medication adherence for children seen at outpatient department from western China, and identify characteristics associated with nonadherence. We conducted a cross-sectional survey. Participants were recruited by consecutive sampling from the outpatient of the West China Second Hospital from October 2021 to April 2022. The Morisky Medication Adherence Scale (MMAS-8) was used to evaluate patients’ medication adherence. A multivariate linear regression model was used to analyze influencing factors. 1206 children with a mean age of 6.02 ± 3.86 years were included. Seventeen percent (208/1206) of patients showed good adherence, 24.7% (298/1206) showed moderate adherence, and 58% (700/1206) showed poor adherence. Thirty-five percent (428/1206) of guardians had anxiety. Factors that influenced medication adherence included anxiety score of guardian (*P* = 0.030), education level of guardian (*P* = 0.003), annual household income (*P* = 0.001), and days the patient is on the medication (*P* = 0.023). A majority of children seen at outpatient department from West China had low medication adherence, and depression and anxiety among guardians were common. Implementing health education measures will be important for improving medication adherence in future.

## Introduction

Medication adherence can be defined as “the extent to which patients take medication with recommendations from a healthcare provider”^1^. Medication adherence refers to the degree to which the medications taken reflect the prescriber’s intention^2^. Poor medication adherence is common, especially in chronic disease, and is associated with worse health outcomes^3^. A systematic review of 10 studies reported that poor medication adherence is related to increased health care use in children who have a chronic disease^4^. Medication adherence is affected by a variety of factors, including the individual basic characteristics of children, children’s family, and communications with healthcare providers^5^.

Despite potential negative impacts on the effectiveness of drug therapy, children generally exhibit a high prevalence of medication non-adherence. Edgcomb et al.^6^ conducted a systematic review and included 28 studies with 180,870 children with severe mental illness, the meta-analysis showed that only 65.9% children were medication adherent. The results of influencing factors presented that patient and family attitudes toward care, adherence to psychotherapy, and insight affected medication adherence. Chloe et al.^7^ assessed medication adherence regarding acne drugs in children and adolescents with acne vulgaris in the United States, and included 20,039 eligible children and adolescents. The results revealed that only 3.71% of children were adherent to acne medication, while 13.38% of adolescents were adherent. The influencing factors included clinical characteristics, age, type of medication, sex, and whether the patient got a refill^7^. Warembourg et al.^8^ evaluated the rate of medication adherence among 75 children with anti-infective drugs prescribed for acute infection at hospital discharge. Only 34.9% of children exhibited good adherence, and parents’ lack of knowledge regarding anti-infective treatment was the main reason for non-adherence^8^. Shamil et al.^9^ used a cross-sectional study design to evaluate antiepileptic drug adherence among 192 children with epilepsy. The results revealed that 65% children were adherent to their medication, and that low family income and occurrence of seizures affected adherence^9^.

In China, Yu et al. evaluated hormone medication adherence in 96 children with chronic kidney disease. The results revealed medication nonadherence in 52% of cases, and that duration of illness, residence and the mother’s educational level were independent factors affecting medication adherence^10^. Yang et al.^11^ examined 204 patients with tic disorder in West China, revealing that only 40.7% of children exhibited good medication adherence, and that decreasing quality of life and living in non-rural areas were significant independent determinants of non-adherence. However, the prevalence of medication adherence and influencing factors reported in previous studies exhibit substantial variation, and the sample sizes of previous studies in China have been relatively small.

In addition, although a previous meta-analysis^12^ revealed that anxiety is common in parents of young people with chronic health conditions, few studies have explored the relationships between guardians’ mental state and children’s medication adherence. Therefore, there is currently a lack of research on medication adherence among children in China. It is necessary to conduct cross-sectional studies to evaluate the prevalence of medication adherence, assess the association between guardians’ mental health and medication adherence for outpatients from western China, and identify characteristics associated with nonadherence.

## Methods

### Study design

We used a cross-sectional study design to assess adherence to prescribed medication for outpatients at the West China Second Hospital of Sichuan University.

### Study population

Participants were recruited by consecutive sampling from outpatients at the West China Second Hospital from October 2021 to April 2022. The inclusion criteria were as follows: (1) under the age of 18 years; (2) taking medication; (3) parents of children voluntarily participated in this study; (4) written informed consent was provided.

Exclusion criteria were as follows: (1) guardians could not understand and complete the questionnaire (i.e., guardians are not familiar with the medication use); (2) children were unwilling to participate in the survey; (3) children had never taken medicine before.

### Sample size calculation

We used the formula (n = u^2^π(1 − π)/δ^2^) for estimation of sample size when estimating the population rate in the sampling survey to calculate the minimum patient sample size. The adherence rate reported for chronically ill children was 83%^13^, assuming precision of 5% (0.05), allowable error (δ) of 0.03, and an available population of patients with 95% confidence limits. The resulting sample size was 603, and considering a 20% loss of follow-up rate, the minimum sample size was 754 patients.

### Survey content

Referring to expert opinions, related literature and clinical experience, we designed the questionnaire and carried out investigation at the outpatient department. Children > 8 years old completed the questionnaire independently, while children < 8 years old were assisted by their guardian. The evaluation of the guardian’s mental status was provided by the guardian themselves.

The questionnaire included four aspects: (1) basic characteristics: gender, age, children’s schooling, children’s residence; (2) guardian’s status: the main guardian, the age of the main guardian, the education level of the guardian, the employment status of the main guardian, the frequency of getting along with each other, and annual family income (According to Statistical Yearbook of Sichuan Province in 2022, per capita total income of urban households is 50,228, we suppose a family has two employees, so the standard is 100,000 yuan); (3) disease and medication status: disease, medication, medication time (the days the patient is on the medication); (4) mental status of the guardian and children’s medication adherence: anxiety, depression, and medication adherence status.

### Outcome measure

#### Mental status

The Generalized Anxiety Disorder-7 (GAD-7) scale was used to measure the status of anxiety^14^. Each item describes one typical generalized anxiety disorder symptom and is evaluated by the frequency with which that symptom was reported over the last 2 weeks. Each item is scored from 0 (not at all) to 3 (nearly every day). Item scores are summed to give a total GAD-7 score ranging from 0 to 21. Scores of 0–4 are defined as no anxiety, scores of 5–9 are defined as mild anxiety, scores of 10–13 are defined as moderate anxiety, scores of 14–18 are defined as moderate to severe anxiety, and scores of 19–21 are defined as severe anxiety.

The 9-item Patient Health Questionnaire (PHQ-9) was used to measure participants’ depression status^15^. The PHQ-9 focuses on the frequency of occurrence over the past 2 weeks of nine depressive symptoms derived from the Diagnostic and Statistical Manual of Mental Disorders, Fourth Edition diagnostic criteria. Each item is scored from 0 (not at all) to 3 (nearly every day), with total scores ranging from 0 to 27. PHQ-9 scores of 0–4 are defined as no depression, scores of 5–9 points are defined as mild depression, scores of 10–14 points are defined as moderate depression, and scores of 20–27 are defined as severe depression.

#### Medication adherence status

We used the Morisky Medication Adherence Scale (MMAS-8) to evaluate patients’ medication adherence. MMAS-8 was widely used in Chinese population, the Chinese version of MMAS-8 is reported to have acceptable internal consistency and has demonstrated construct validity^16^. We attended a training and certification session for the Morisky Widget in August 2019 in Beijing, China, and obtained licenses for the use of MMAS-8 from MMAS Research LLC, USA. The full score of the MMAS-8 is 8, with a score of < 6 indicating low adherence, a score of 6–7 indicating medium adherence, and a score of 8 indicating high adherence^17–19^.

#### Quality control

Before the formal investigation, we selected the participants in the outpatient department for pre-investigation. During the investigation, a professional investigator who had received standardized training explained the purpose, significance, and content of this investigation to each participant. After obtaining informed consent, the investigation was conducted face to face. After the questionnaires were collected, the quality of all collected questionnaires was reviewed by a professionally trained researcher. For unqualified questionnaires, such as those with missing items and contradictory answers, telephone return visits were used to supplement and improve questionnaire responses.

### Data analysis

SPSS version 22 (SPSS Inc., Chicago, IL, USA) was used for statistical analysis. Quantitative data were expressed as mean and standard deviation, and t-tests or ANOVA analysis were used. Qualitative data were counted by composition ratio, and chi-square tests were used. Following a univariate analysis, factors with a *P*-value ≤ 0.1 were included in the multivariate linear regression model. *P*-values ≤ 0.05 were considered to be statistically significant.

The study was approved by the Office of Research Ethics Committees of West China.

Second Hospital. Written informed consent was obtained from all caregivers, and consent was also obtained from children aged > 8 years.

### Ethical approval

This study was performed in line with the principles of the Declaration of Helsinki. The study was approved by the Office of Research Ethics Committees of West China Second Hospital.

### Consent to participate

Written informed consent was obtained from all caregivers, and consent was also obtained from children aged > 8 years.

## Results

### The characteristics of included participants

A total of 1206 children were included, with a mean age of 6.02 ± 3.86 years. Characteristics of the included participants are summarized in Table 1.

**Table 1:** Demographics and baseline patient characteristic.

Item	Option	n	%	Score of adherence	F/t	*P*
Gender	Male	606	50.2	5.18 ± 2.05	− 0.113	0.910
	Female	600	49.8	5.19 ± 2.08		
Age (years)	6.02 ± 3.86				− 0.112	0.337
School attendance	Not attending school	352	29.2	5.17 ± 2.12	0.013	0.909
	Attending school	854	70.8	5.19 ± 2.04		
Residence	City	845	70.1	5.17 ± 2.08	− 0.528	0.597
	Non-city	361	29.9	5.24 ± 2.09		
Guardian	Parents	845	70.1	5.17 ± 2.06	− 0.528	0.597
	Non-parents	361	29.9	5.24 ± 2.08		
Age of guardian (years)	35.69 ± 8.24				1.479	0.140
Education level of guardian	High school and below	438	36.3	5.39 ± 2.01	2.549	0.011
	University or above	768	63.7	5.07 ± 2.09		
Working status of guardian	At work	790	65.5	5.10 ± 2.11	− 2.000	0.046
	Not at work	416	34.5	5.35 ± 1.97		
Frequency of interaction	Get along every day	1138	94.4	5.18 ± 2.06	− 0.242	0.809
	Not getting along every day	68	5.6	5.25 ± 2.16		
Annual household income (yuan)	Below 100,000 yuan	745	61.8	5.07 ± 2.03	− .2.499	0.013
	Over 100,000 yuan	461	38.2	5.38 ± 2.10		
Disease system	Respiratory system diseases	415	34.4	5.20 ± 2.20	0.876	0.556
	Digestive system diseases	204	16.9	5.00 ± 2.08		
	Neuromuscular system diseases	282	23.4	5.26 ± 2.03		
	Hematological system disease	20	1.7	5.05 ± 2.30		
	Urinary system diseases	26	2.2	5.31 ± 1.86		
	Nutritional disorders	59	4.9	5.21 ± 2.05		
	Immunological diseases	40	3.3	5.26 ± 1.99		
	Infectious diseases	27	2.2	5.01 ± 1.49		
	Cardiovascular diseases	21	1.7	6.29 ± 1.24		
	Hereditary diseases	19	1.6	5.24 ± 2.22		
	Other	93	7.7	5.05 ± 1.77		
Classification of medication	Antipyretic-analgesic and anti-inflammatory drugs	136	11.3	4.67 ± 2.24	2.289	0.012
	Antibiotics and other anti infective drugs	106	8.8	5.53 ± 2.05		
	Drugs for improving digestive function	98	8.1	5.13 ± 2.00		
	Nutritional drugs such as vitamins and calcium	334	27.7	5.22 ± 2.07		
	Cough relieving, asthma relieving, and expectorant drugs	171	14.2	5.48 ± 2.05		
	Antitumor drugs	4	.3	4.88 ± 2.17		
	Drugs for improving renal function	8	.7	5.53 ± 2.15		
	Psychotropic drugs such as antiepileptic and anticonvulsant drugs	23	1.9	6.12 ± 2.17		
	Immunomodulatory drugs	34	2.8	5.37 ± 1.98		
	Drugs for improving cardiac function	7	.6	5.18 ± 2.10		
	Other	285	23.6	5.01 ± 1.94		
Medication time	Less than 1 week	702	58.2	5.10 ± 2.13	3.058	0.047
	1 week to 3 months	236	19.6	5.13 ± 1.93		
	Over 3 months	268	22.2	5.46 ± 1.99		
Anxiety score of guardian (score)	3.63 ± 4.27				− 4.195	< 0.001
Depression score of guardian (score)	4.67 ± 4.90				− 3.838	< 0.001

F or t is the Statistical value for ANOVA analysis or t test, respectively. Disease system means the diseases the patients have.

### Medication adherence rates

Overall, 17.2% (208/1206) of patients showed good adherence, 24.7% (298/1206) showed moderate adherence, and 58% (700/1206) showed poor adherence.

### Prevalence of anxiety and depression of guardian

The anxiety scores of guardian ranged from 0 to 24, with a mean score of 3.63 ± 4.271. Overall, 64.51% (778/1206) of guardians had no anxiety, 26.37% (318/1206) had mild anxiety, 5.31% (64/1206) had moderate anxiety, and 3.82% (46/1206) had moderate to severe anxiety.

Depression scores ranged from 0 to 27, with a mean score of 4.67 ± 4.902; 57.6% (695/1206) had no depression, 28.6% (345/1206) had mild depression, 8.9% (107/1206) had moderate depression, and 4.9% (59/1,206) had moderate to severe depression.

### Factors affecting medication adherence (Tables 1, 2)

**Table 2:** Multiple linear regression analysis of factors influencing medication adherence.

Variable	Non-standardized coefficient	Standard error	Standard coefficient	t	*P*
Constant	4.992	0.368		13.582	0.000
Anxiety score of guardian	− 0.046	0.021	− 0.096	− 2.173	0.030
Depression score of guardian	− 0.017	0.019	− 0.041	− 0.941	0.347
Education level of guardian	− 0.401	0.133	− 0.093	− 3.012	0.003
Working status of guardian	0.228	0.128	0.053	1.788	0.074
Annual household income	0.420	0.128	0.099	3.283	0.001
Classification of medication	− 0.010	0.017	− 0.018	− 0.614	0.539
Medication time	0.164	0.072	0.065	2.276	0.023

Seven variables had statistically significant associations with medication adherence scores in the univariate analysis: education level of guardian (*P* = 0.011), working status of guardian (*P* = 0.046), annual household income (*P* = 0.013), classification of medication (*P* = 0.012), medication time (*P* = 0.047), anxiety score of guardian (*P* < 0.001) and depression score of guardian (*P* < 0.001). And we also used the multiple comparison method of LSD to compare the medication adherence in different classification of medication and medication time. For the medication groups, number of comparisons is 11*(11 − 1)/2 = 55, and corrected *P*-value is 0.05/55 = 0.0009. For the medication time groups, number of comparisons is 3*(3 − 1)/2 = 3, and corrected *P*-value is 0.05/3 = 0.017. The results showed there statistically significance in one comparison: Less than 1 week vs Over 3 months, *P* = 0.015.

The multiple linear regression model revealed that lower anxiety score of guardian (β =  − 0.096; SE = 0.021; *P* = 0.030), higher education level of guardian (β =  − 0.093; SE = 0.133; *P* = 0.003), higher annual household income (β = 0.099; SE = 0.128; *P* = 0.001) and longer medication time ($\beta$ = 0.065; SE = 0.072; *P* = 0.023) were associated with high medication adherence scores.

## Discussion

This cross-sectional survey evaluated medication adherence among children seen at outpatient department in west China, and analyzed factors influencing medication adherence. We used MMAS-8 to assess the medication adherence, which included 8 items and could affect the medication adherence in the past weeks, the main reasons for non-adherence were as follows: (1) patients or their guardian have forgotten to use medication; (2) patients or their guardian have adjusted or stopped medication when the condition is aggravated or changed. The results showed that 58% children had poor adherence, which was higher than the proportion previously reported in chronically ill children from Malaysia (28.4%)^13^, and that in children with inflammatory bowel disease in Korea (33.2%)^20^. There are several possible reasons for this discrepancy, as discussed below. (1) West China Second Hospital is one of the largest children’s hospitals in west China, and undertakes a substantial amount of clinical work, including treatment, referral and consultation of critically ill women and children in Chengdu, Sichuan Province, and Southwest China. The diagnosis and treatment level for many diseases in children is high in China, so children admitted to the hospital often have serious diseases and are difficult to treat, which may result in poor adherence. (2) The medication time for the majority of included patients was less than 1 week. It is possible that patients and their guardians were not familiar with the methods and requirements of medicine use, leading to poor medication adherence. However, the current adherence rates were similar to those previously reported for anti-infective medication adherence in children seen at outpatient department in France (65.1%)^8^, and those reported in a meta-analysis of the prevalence rates of medication adherence to antiepileptic drugs (58%)^21^. In the current study, the most common patient diseases were respiratory system diseases, digestive system diseases, and neuromuscular system diseases. There may be some similarities in the use of drugs for these various diseases, leading to similar adherence rates of medication. Additionally, we found that anxiety and depression were relatively common in guardians, possibly because the study was conducted during the spread of the coronavirus disease epidemic in China. Fear among patients and their guardians related to the epidemic may have affected their rates of anxiety and depression.

Regarding the factors influencing medication adherence, we found that lower education level of guardian, higher annual household income, and longer medication time were associated with higher medication adherence scores, which was similar to the results of previous studies^22–24^. Because family with better economic conditions tend to have better health literacy and are more familiar with methods for the use and timing of drugs, their medication adherence may be better. The timing of taking medicine is also an important factor affecting adherence, and patients may know more about the use of drugs. The current results indicated that lower anxiety scores of guardians predicted medication adherence, which was a novel finding in the present study. In addition, we found a higher guardian’s educational level and a higher household income have the opposite effect on medication adherence, the reason may be as follows: (1) 29.9% guardians were non-parents in our study, who have high educational level, but because they have retired, it doesn't mean that the family income level is high. (2) People with higher educational level are more likely to trust their own judgment or experience, and less likely to listen to doctors' advice. The prevalence of anxiety among guardians in this study was higher than that reported in a meta-analysis of anxiety in parents of young people with chronic health conditions (16%)^12^. However, the relationship between anxiety and treatment adherence has received little research attention^25^.

Therefore, in clinical practice, it is important to develop health education sessions for children with chronic diseases and their guardians to improve adherence in children. The content of health education should be diversified and multi-dimensional, including knowledge of chronic diseases, methods of taking drugs, dosage, treatment duration, and any other issues requiring attention^26^. Additionally, some evidence suggests that adherence can be improved by applying specific communicative consultation skills, highlighting the importance of conducting close follow-up of patients with chronic diseases to establish a therapeutic partnership with the family, which can promote constructive and collaborative dialogue between the medical team and the family^27^.

At present, there are many methods to measure medication adherence, including subjective measurement (i.e., Morisky scale) and objective measurement (therapeutic drug monitoring (TDM), electronic devices, and pickup/refill rates), each method has its advantages and disadvantages^28–30^. In the studies of medication adherence of various diseases, there was a wide range of medication adherence, explained largely by the heterogeneity of assessment methods. Objective measurement could quantitatively calculate the adherence, and the conclusion is relatively accurate. However, TDM is expensive and it is difficult for patients to accept high frequency. For the method of pickup/refill rates, medication Possession Ration (MPR) is defined as the proportion (or percentage) of days’ supply obtained over either refill interval, where last refill is the end point, or fixed refill, where a specific time period is set^31^. MPR is a very simple calculation method which does not consider the gaps in refills and the need for continuous therapy with multiple prescriptions. Consequently, overestimated adherence values are found while using this method^32^. Proportion of days covered (PDC) is defined as the total days’ supply was divided by number of days of study participation^33^, patients are required to keep medical records or medication records completely^30^, it may be difficult for some children. Although we did not used some objective measures to assess the medication adherence, many studies produced moderate to high correlation between both self-reported questionnaires (SRQs) and monitoring devices^34^. As there is not yet a ‘gold standard’ measure for monitoring patient adherence^34^, SRQs and Medication Event Monitoring Systems operating together continue to emerge as the preferred effective method for measuring medication adherence.

Our study involved several limitations. First, we did not use random sampling to select children as participants. Second, our study used a single-center design. However, the sample size of this study was relatively large, and West China Second Hospital accepts patients from various provinces in western China, providing a representative sample. Third, the assessment of medication adherence and mental health was based on self-reporting of patients or caregivers, and the findings might therefore not reflect their actual practice. So multiple methods assessment could be used in the future, such as self-report questionnaires, structured interviews, TDM, electronic devices, and pickup/refill rates. Fourth, this proportion of the education level of university or above is high, because 70.1% participants were from the Chengdu which is the capital city of Sichuan and a new first-tier city in China, with good economic and educational development, so our result may overestimate or underestimate the relevant outcome measures. Future research should be conducted in multi-centers and used various adherence measurement methods, which could address these limitations.

In conclusion, A majority of children seen at outpatient department from West China had low medication adherence, and depression and anxiety among guardians were common. It will be necessary to implement health education measures to improve medication adherence.

## Data Availability

According to the data confidentiality agreement of the corresponding author, the data is not disclosed, but is stored in the secured computer, which can be obtained by the corresponding author and used for scientific research purposes only.

## Abbreviations

GAD-7: The generalized anxiety disorder-7
PHQ-9: The 9-item patient health questionnaire
MMAS-8: Morisky medication adherence scale

## Code availability

Software application used in the study Excel.

## References

[1] Osterberg L, Blaschke T (2005). Adherence to medication. N. Engl. J. Med..

[2] Hartman L, Lems WF, Boers M (2019). Outcome measures for adherence data from a medication event monitoring system: A literature review. J. Clin. Pharm. Ther..

[3] Fernandez-Lazaro CI (2019). Medication adherence and barriers among low-income, uninsured patients with multiple chronic conditions. Res. Soc. Adm. Pharm..

[4] McGrady ME, Hommel KA (2013). Medication adherence and health care utilization in pediatric chronic illness: A systematic review. Pediatrics..

[5] McQuaid EL, Landier W (2018). Cultural issues in medication adherence: Disparities and directions. J. Gen. Intern. Med..

[6] Edgcomb JB, Zima B (2018). Medication adherence among children and adolescents with severe mental illness: A systematic review and meta-analysis. J. Child Adolesc. Psychopharmacol..

[7] Hester C (2016). Medication adherence in children and adolescents with acne vulgaris in medicaid: A retrospective study analysis. Pediatr. Dermatol..

[8] Warembourg M (2020). Assessment of anti-infective medication adherence in pediatric outpatients. Eur. J. Pediatr..

[9] Dima SA, Shibeshi MS (2022). Antiepileptic drug adherence in children in southern Ethiopia: A cross sectional study. PLoS One.

[10] Yu L, Tang YX, Yu M, Zhou QL, Wang T (2016). Analysis on the influencing factors for hormone medication compliance in children with chronic kidney disease. West China Med. J..

[11] Yang CS, Qin WX, Yu D, Li JY, Zhang LL (2019). Medication adherence and associated factors for children with tic disorders in Western China: A cross-sectional survey. Front. Neurol..

[12] Pinquart M (2019). Meta-analysis of anxiety in parents of young people with chronic health conditions. J. Pediatr. Psychol..

[13] Chew CC, Chan HK, Chang CT, Hss AS, Hassali MA (2021). Medication-related knowledge, administration practice and adherence among caregivers of chronically ill children in Malaysia. BMC Pediatr..

[14] Tong X (2016). Validation of the generalized anxiety disorder-7 (GAD-7) among Chinese people with epilepsy. Epilepsy Res..

[15] Kroenke K, Spitzer RL, Williams JB (2001). The PHQ-9: Validity of a brief depression severity measure. J. Gen. Intern. Med..

[16] Ho CP, Lee TJF (2014). The reliability and validity of the Chinese version of the eight-item Morisky medication adherence scale. Value Health.

[17] Morisky DE, Ang A, Krousel-Wood M, Ward HJ (2008). Predictive validity of a medication adherence measure in an outpatient setting. J. Clin. Hypertens (Greenwich).

[18] Krousel-Wood M (2009). New medication adherence scale versus pharmacy fll rates in seniors with hypertension. Am. J. Manag. Care.

[19] Morisky DE, DiMatteo MR (2011). Improving the measurement of self reported medication nonadherence: Response to authors. J. Clin. Epidemiol..

[20] Lim JK, Lee YJ, Park JH (2020). Medication related knowledge and medication adherence in in pediatric and adolescent patients with inflammatory bowel disease. J. Korean Med. Sci..

[21] Yang CS, Hao ZL, Yu D, Xu QF, Zhang LL (2018). The prevalence rates of medication adherence and factors influencing adherence to antiepileptic drugs in children with epilepsy: A systematic review and meta analysis. Epilepsy Res..

[22] Lor M, Koleck TA, Bakken S, Yoon S, Navarra AD (2019). Association between health literacy and medication adherence among hispanics with hypertension. J. Racial Ethn. Health Dispar..

[23] Yang CS, Yu D, Li JY, Zhang LL (2020). Prevalence of medication adherence and factors influencing adherence to antiepileptic drugs in children with epilepsy from western China: A cross-sectional survey. Epilepsy Behav..

[24] Smita S (2021). Assessment of adherence to medication during chronic illnesses in pregnancy. Perspect. Clin. Res..

[25] Volpato E, Toniolo S, Pagnini F, Banfi P (2021). The relationship between anxiety, depression and treatment adherence in chronic obstructive pulmonary disease: A systematic review. Int. J. Chron. Obstruct. Pulmon. Dis..

[26] Da Mota GM, Navarro T, Keepanasseril A, Jeffery R, Haynes RB (2017). Increasing adherence to treatment in epilepsy: What do the strongest trials show?. Acta Neurol. Scand..

[27] Brand PL, Klok T, Kaptein AA (2013). Using communication skills to improve adherence in children with chronic disease: The adherence equation. Paediatr. Respir. Rev..

[28] Hoegy D (2019). Medication adherence in pediatric transplantation and assessment methods: A systematic review. Patient Prefer Adher..

[29] Davies G (2020). Methods for assessment of patient adherence to removable orthoses used after surgery or trauma to the appendicular skeleton: A systematic review. Trials..

[30] Ma LL (2016). Review of compliance assessment methods. Chin. Pharm. Aff..

[31] Andrade SE, Kahler KH, Frech F, Chan KA (2006). Methods for evaluation of medication adherence and persistence using automated databases. Pharmacoepidemiol. Drug Saf..

[32] Lam WY, Fresco P (2015). Medication adherence measures: An overview. Biomed. Res. Int..

[33] Hess LM, Raebel MA, Conner DA (2006). Measurement of adherence in pharmacy administrative databases: A proposal for standard definitions and preferred measures. Ann. Pharmacother..

[34] Monnette A, Zhang Y, Shao H, Shi L (2018). Concordance of adherence measurement using self reported adherence questionnaires and medication monitoring devices: An updated review. Pharmacoeconomics.

